# Refractory Status Epilepticus Responsive to Electroacupuncture at Shuigou Acupoint: A Case Report

**DOI:** 10.3389/fneur.2020.580777

**Published:** 2020-11-30

**Authors:** Fang Yuan, Aili Lu, Shibiao Wu, Lixin Wang

**Affiliations:** The Second Affiliated Hospital of Guangzhou University of Chinese Medicine, Guangzhou, China

**Keywords:** refractory status epilepticus, non-convulsive status epilepticus, electroacupuncture, critical care, case report

## Abstract

Refractory status epilepticus (RSE) is a critical and intractable neurological emergency. Around 55% of RSE episodes still persist despite high dose of continuous infusion of anesthetics. It's a clinical urgency and challenge to search for novel alternative treatments to control RSE as soon as possible. Here, we reported a case of RSE in a 67-year-old woman with varicella-zoster virus encephalitis. She had persistent non-convulsive SE despite the continuous infusion of midazolam. On the basis of fundamental treatments, she was given electroacupuncture at Shuigou acupoint for 10 min. An immediate EEG suppression was seen after the electroacupuncture treatment and lasted for 9 min, and lasting epileptic discharges (> 10 s) and clinical seizures were not observed any more. Midazolam was withdrawn gradually 24 h later. This case report may bring an alternative treatment for RSE.

## Introduction

Status epilepticus (SE) is acommon life-threatening neurological emergency, and especially the mortality of generalized convulsive SE (GCSE) and non-convulsive SE (NCSE) in coma can be as high as 60% (1–3). After the first-line treatment, about 30–40% of SE episodes still persist and require intravenous antiepileptic drugs (AEDs), such as valproate and levetiracetam (4, 5). Up to 45% of SE episodes cannot be resolved even after adequate administration of benzodiazepines and at least one intravenous AED (6), which are labeled refractory SE (RSE). Patients with RSE require continuous infusion of anesthetics (CIVADs) under close monitoring of vital signs and EEG. Unfortunately, 55% of RSE episodes still persist despite high dose of CIVADs (6). The lasting influence of SE aggravates neuronal injury and increases mortality. It is urgent to search for effective and multidimensional therapies to terminate RSE as soon as possible.

Acupuncture has a long history of treating seizures in China. Modern studies indicated that electroacupuncture could reduce neuronal excitability in rats with epilepsy by enhancing the activity of nitric oxide synthase and superoxide dismutase (7), modulating the expression of neuropeptides (e.g., leu-enkephalin, dynorphin, and cholecystokinin) (8, 9), and regulating the expression of GAD67mRNA (10). A recent study in rats with SE induced by kainic acid demonstrated that electroacupuncture at Shuigou (DM26) acupoint enhanced the expression of GAD 67 and Glutamate Transporter and reduced seizure activity (11). For non-refractory SE that can be easily controlled by benzodiazepines and intravenous AED, alternative treatments like acupuncture are not very necessary. However, the control of RSE has always been a clinical challenge calling for novel effective interventions. For the first time, we tried electroacupuncture in a patient with RSE and surprisingly found that electroacupuncture terminated RSE rapidly, without return of seizures. Here, we reported this case in detail.

## Case Presentation

### Clinical History

A 67-year-old woman was admitted to our neurological intensive care unit (NICU) because of coma and skin rashes for 1 day. She had a fever of 38°C with fatigue, dizziness, and muscle soreness 3 days before admission. On the afternoon of admission day, she developed continuous twitches of face and jerking movements of left arm and leg.

### Clinical Examination and Diagnosis

Her Glasgow Coma Scale (GCS) was 5 (E1V1M3). The sizes of two pupils were different: left was 2.5 mm, right was 3.5 mm. Direct light reflexes of both pupils were slow, corneal reflexes of both sides were present. Four limbs had increased muscle tone and flexed mildly to painful stimuli. Deep tendon reflexes in all her limbs were diminished. Bilateral Babinski signs, Chaddock signs, Oppenheim signs, Gordon signs, and Hoffman's signs were all negative. Her neck was not rigid, and Brudzinski's sign was negative. Her cerebrospinal fluid (CSF) opening pressure was 250 mm H_2_O. CSF analysis was significant for: glucose was 8.13 mmol/L (concurrent serum glucose was 14.43 mmol/L); chloride, 114.7 mmol/L; protein, 12,782 mg/L; red blood cell count, 1,410 × 10^6^/L; white blood cell (WBC) count, 480 × 10^6^/L; neutrophil (NEUT) in percentage, 30.0%, lymphocytes (LYM) in percentage, 64.0%; monocyte (MONO) in percentage, 6.0% (concurrent serum test: WBC count, 4.47 × 10^9^/L; NEUT, 65.5%; LYM%, 25.1; MONO%, 9.2). Metagenomic next-generation sequencing testing of CSF indicated varicella-zoster virus (VZV). Based on negative bacterial culture, fungal and mycobacterial staining results, she was diagnosed with VZV encephalitis.

### Neuroimaging

**Figure 1:**
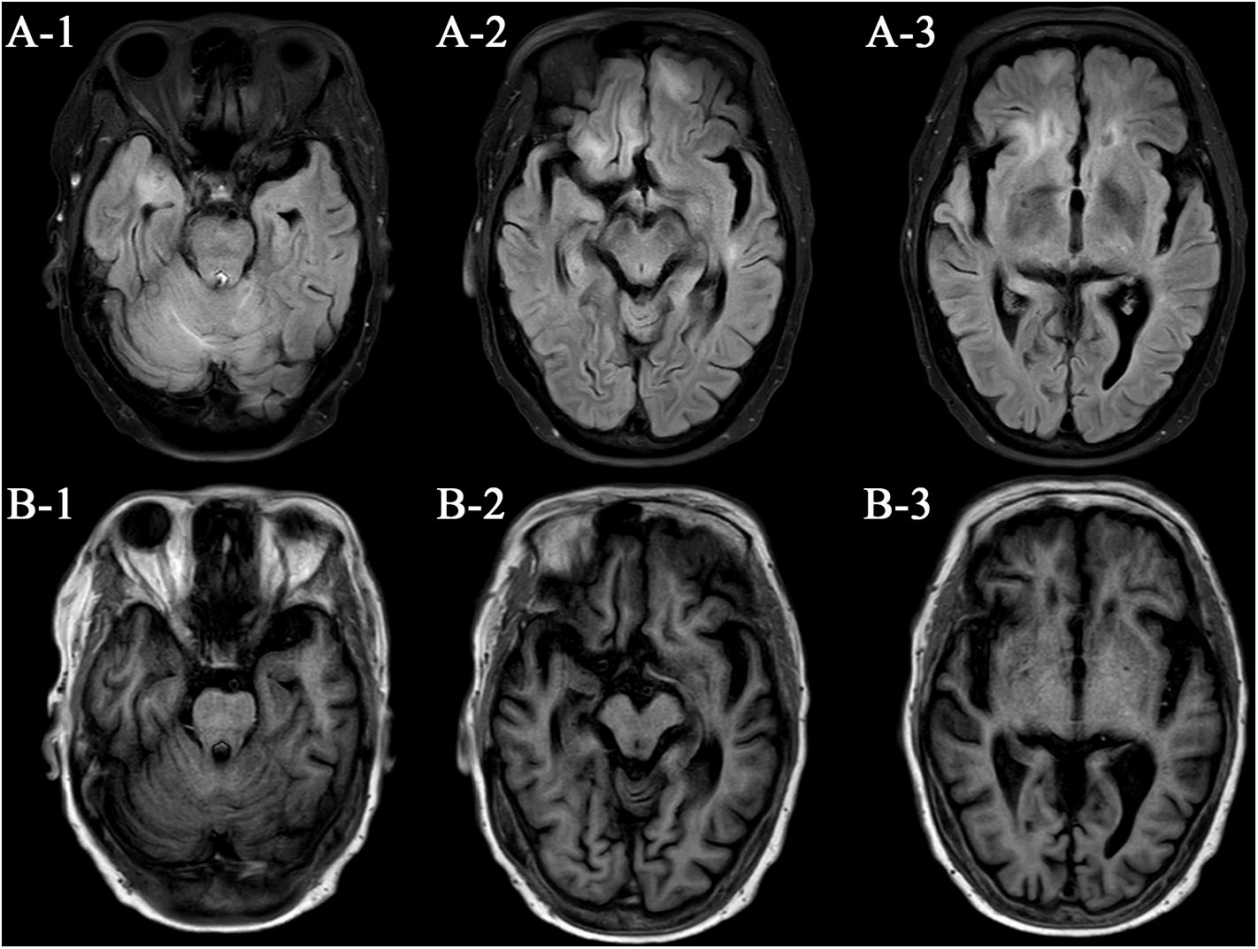
Neuroimaging presentation. **(A)** Flair images; **(B)** T1 images.

Lesions with high T2 and FLAIR signal were seen in right temporal lobe and bilateral frontal lobes (Figure 1). Gadolinium enhancement was presented in pia mater near prepontine cistern and cerebellar hemispheres, and in endocranium near frontal and temporal lobes. Microbleeds were found in bilateral thalami and coronae radiatae.

### Continuous Video-EEG Monitoring and SE Treatment

This patient was suffering from continuous subtle motor twitches in the left eyelid and left face, and lasting polyspike activities were seen on her scalp EEG (Fp2, F4, F7, F8). Her skin rashes, neurological symptoms, and MRI characteristics suggested a possibility of herpes-zoster virus infection (12, 13). Therefore, acyclovir (600 mg, iv, q8h) and dexamethasone (10 mg, iv, qd) were administered immediately after admission. Besides treatments for the etiology of SE, two doses of diazepam 10 mg were given intravenously 5 min apart, with no immediate clinical or electrographic changes. An intravenous bolus of valproate 1,800 mg was then administered as the second-line therapy, but SE still persisted. An intravenous bolus of midazolam 12 mg was given 30 min after the use of valproate, then a continuous infusion of midazolam was used. However, her SE was still lasting despite the infusion rate of 0.4 mg/kg/h which is the maximal infusion rate of midazolam recommended by Chinese consensuses on SE (14).

### Electroacupuncture Treatment

**Figure 2:**
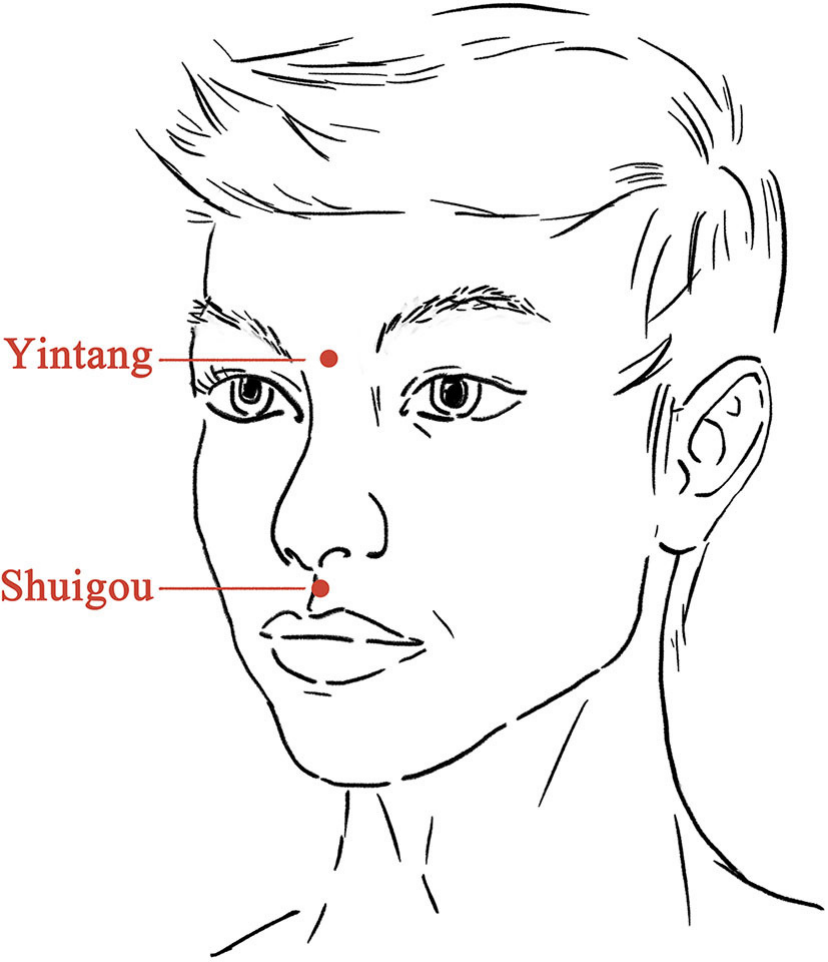
Location of acupoints for the electroacupuncture.

Electroacupuncture treatment was given 6 h after SE onset and 4 h after the use of midazolam. After skin disinfection, one steel needle (0.25 × 40 mm, Hwato, China) was inserted at Shuigou (DM26, on the face, at the junction of the superior 1/3 and middle 1/3 of the philtrum) pointing to the nose ~10 mm into the skin with an angle of 30°, and the other needle was inserted at Yintang (at the glabella, at the midpoint between the medial extremities of the eyebrows) pointing to the nose ~20 mm into the skin with an angle of 15° (Figure 2). Paired electrodes from the electroacupuncture device (Xinsheng, G6805-I, China) were placed on the needles at Shuigou and Yintang. A continuous current (50 Hz, 10 mA) was given for 10 min.

### Treatment Response

**Figure 3:**
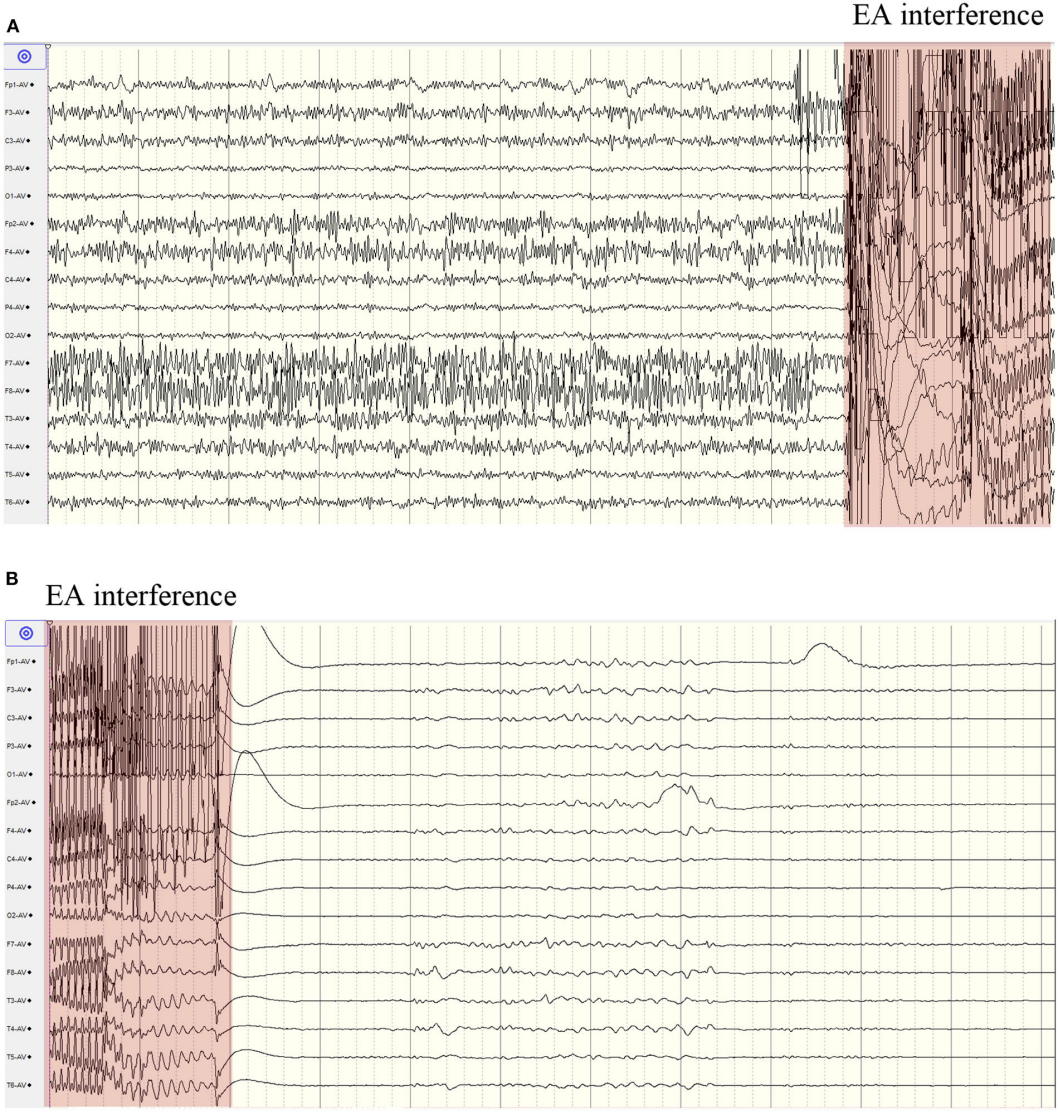
Continuous scalp EEG recording before and after electroacupuncture. **(A)** epileptic discharges before electroacupuncture; **(B)** EEG suppression after electroacupuncture. EA, electroacupuncture.

The twitches of her eyelids and face ceased 6 min after the start of electroacupuncture treatment. During the 10 min of electroacupuncture treatment, the EEG was unable to be analyzed due to great interference caused by electroacupuncture. Immediately after the end of electroacupuncture treatment, a period of suppression was seen on EEG, and EEG seizures were controlled (Figure 3). All her vital signs were stable during the electroacupuncture treatment. The suppression pattern lasted for around 9 min, then diffuse slow waves gradually appeared. Sporadic epileptic discharges were seen on the right temporal scalp electrodes, but lasting epileptic discharges (> 10 s) and clinical seizures were not observed. Midazolam was withdrawn gradually 24 h later, and EEG monitoring was discontinued 70 h later. One day after the withdrawal of midazolam, she opened eyes to painful stimuli. Three days later, she opened eyes to shouted requests. Seven days later, she opened eyes spontaneously and was able to obey motor commands, and her skin rashes were diminished.

## Discussion

As RSE lasts longer and longer, the treatment effect declines, the comorbidity develops, and the mortality climbs (15, 16). Continuous intravenous infusion of anesthetic drugs is the primary treatment for RSE which is refractory to intravenous benzodiazepines and AEDs. Acting on gamma-aminobutyric acid (GABA) receptor, midazolam and propofol are the most widely used anesthetic agents for RSE. Several cases series studies reported that ketamine, N-methyl-D-aspartate (NMDA) receptor antagonist, might be a relatively effective and safe treatment for RSE (17, 18). Besides, the efficacy of ketogenic diet, immunomodulator, hypothermia, vagus nerve stimulation, deep brain stimulation, electroconvulsive therapy, and corpus callosotomy on RSE were also studied by a few case studies. However, rapid controlling of RSE remains a clinical challenge. Compared to above therapies, the treatment of electro-acupuncture is relatively safe, economical, convenient, and operable. In this case report, we found that electroacupuncture at DM26 acupoint rapidly controlled RSE which was refractory to continuous infusion of midazolam.

A few case reports suggested that ECT reduced seizures in patients with RSE via generating electrical stimuli with a high intensity (800–900 mA) at a frequency of 60–70 Hz for 1 to 5 s (19–22). ECT was initially used in patients with refractory psychiatric diseases. The anti-epileptic mechanisms of ECT was poorly understood. Studies from patients with major depression indicated that ECT activated endogenous GABAergic pathways and reduced neuronal activity in selected cortical regions (23, 24), which probably can exert an anti-epileptic effect. ECT was reported to be used after a long time from the SE onset (9–103 days), probably because of concerns for possible adverse effects, such as permanent memory changes and confusion (25).

The adverse effects of electroacupuncture were rarely reported in randomized clinical trials (26, 27). The safety and easy operation of electroacupuncture prompt an earlier intervention in RSE. The earlier intervention in SE, the easier would be the control, and the less excitotoxic damage to neurons. The selection of electrostimulation sites may also influence the therapeutic effect. Previous studies showed lower epileptiform activities following ECT (21, 22), whereas a flat recording was observed immediately after electroacupuncture in our study. According to traditional Chinese medicine, Shuigou acupoint belongs to Du meridian which connects to the brain, and it is believed to have the capacity of dredging meridians and awakening consciousness. Shuigou tops the list of “thirteen ghost acupoints” which were used for emergency treatments. Throughout the history many Chinese medical books have recorded the use of Shuigou in epileptic seizure. Modern studies indicated that electroacupuncture at Shuigou controlled SE by enhancing the expression of GAD 67 and Glutamate Transporter (11), and reduced recurrent seizures by inhibiting the neurogenesis in rat dentate gyrus (28).

In conclusion, we presented an RSE case responsive to electroacupuncture at Shuigou acupoint, and no adverse effects were observed. Our finding may bring an alternative treatment for this critical and intractable condition. Future case-series and case-control studies are needed to validate the efficacy of electroacupuncture on RSE and search for the optimal stimulation intensity, stimulation duration, and stimulation intervals.

## Data Availability Statement

The raw data supporting the conclusions of this article will be made available by the authors, without undue reservation.

## Ethics Statement

The studies involving human participants were reviewed and approved by The ethics committee of The Second Affiliated Hospital of Guangzhou University of Chinese Medicine. The patients/participants provided their written informed consent to participate in this study. Verbal and written consent for publication was obtained from patient's legal representative.

## Author Contributions

FY: study concept, design, electroacupuncture treatment, drafting of the manuscript, and study supervision. AL: acquisition, analysis, and interpretation of clinical data. SW: EEG examination and interpretation. LW: study concept, design, and critical revision. All authors contributed to the article and approved the submitted version.

## Conflict of Interest

The authors declare that the research was conducted in the absence of any commercial or financial relationships that could be construed as a potential conflict of interest.
